# Health services for substance use disorders: challenges and future perspectives

**DOI:** 10.1186/s12913-023-10072-y

**Published:** 2023-10-05

**Authors:** João Pedro Silva

**Affiliations:** 1 Associate Laboratory i4HB - Institute for Health and Bioeconomy, Laboratory of Toxicology, Faculty of Pharmacy, University of Porto, Porto, 4050-313 Portugal; 2 UCIBIO, Laboratory of Toxicology, Department of Biological Sciences, Faculty of Pharmacy, University of Porto, Porto, 4050-313 Portugal

## Abstract

Research on the health services’ response to substance use disorders and respective comorbidities holds major relevance due to the increasing prevalence of these ailments. We thus invite contributions to a new Collection of articles launched by BMC Health Services Research titled “Health services for substance use disorders”.

Substance use disorders (SUDs) represent a complex pattern of symptoms associated with the recurrent use of legal (e.g., alcohol, tobacco) and illicit substances, and medications, despite the harmful consequences perceived by such use. Indeed, individuals with SUDs struggle to cope with their daily life obligations and present problems with social and interpersonal interactions [1]. According to data from the Institute of Health Metrics and Evaluation (IHME, University of Washington, WA, USA), the global prevalence of SUDs in 2019 was 2.2% [2]. Moreover, the Substance Abuse and Mental Health Services Administration (SAMHSA) recently reported that 16.5% of the US population aged 12 or older (about 46.3 million people) was diagnosed with a SUD in 2021 [3]. Of note, SUDs are often associated with mental health disorders (e.g., depression, schizophrenia, anxiety, attention-deficit hyperactivity disorder). Indeed, not only does substance use often underlies the onset of mental health disorders, but mental health conditions are known to increase the risk of developing SUDs. Together with the increasing prevalence of SUDs, this concomitant occurrence of mental health-related co-morbidities represents a global concern and a major burden for health services.

The most recent report of the United Nations Office on Drugs and Crime (UNODC) estimated that only 20% of people with SUDs worldwide received treatment in 2021 [4]. This same report also highlighted inequalities in the access to SUD treatment services, as well as in the type and quality of such treatment. In some cases, stigmatization (either self, public, or structural) and discrimination represent a violation of the human rights of people with SUDs and often interfere with these individuals’ choice of seeking treatment or with their adherence to the treatment [5]. Gender inequalities are also visible, as women seem to face more barriers than men to treatment services worldwide. For example, in 2021, only 27% of individuals receiving treatment for amphetamine-type stimulant use were women, despite 45% of all the individuals using these substances being women [4]. Nevertheless, biological sex differences (e.g., endocrine, metabolic, neuronal processes) often determine the individual’s response to a specific treatment. In particular, women with SUDs have been reported to be more vulnerable to family and social relationships’ impairment than men with the same conditions [6]. Still, gender-targeted treatments for SUDs remain particularly scarce [7].

Forcibly displaced populations (e.g., due to natural disasters, or armed conflicts) are also particularly vulnerable to substance use, and present a high risk of developing mental health and substance use disorders [4]. However, the delivery of adequate SUD treatment services in humanitarian settings is often hampered by limited health infrastructures, constrained social and economic resources, as well as by reduced knowledge about the implementation, effectiveness, and evaluation of SUD treatment services in such a context (e.g., due to the heterogeneity and missing information on the patterns of substance use among displaced populations). Data from a recent UNODC-promoted consultation with SUD treatment program administrators, policymakers, service providers, and researchers evidenced the need to improve operational guidance and advance epidemiological, intervention, and implementation research, to ameliorate the access to SUD health services in humanitarian emergencies [8].

Moreover, although evidence-based treatments (e.g., medication-assisted treatment, psychosocial therapies) exist for SUDs, these are not commonly practiced, as only about 25% of health services were estimated to provide such evidence-based treatments for individuals with SUDs [9]. The advances in research on evidence-based treatments for SUDs are evolving at a fast pace. For example, clinical trials addressing the use of psychedelics to treat mental health and SUDs (e.g., psylocybin-assisted therapy) encompass great expectations in this research field. However, these developments may be also associated with a risk of these substances being perceived as “safe”, which might ultimately further increase substance use [4, 10].

Notably, the COVID-19 pandemic represented a major setback to SUD-related health services, not only by contributing to an increase in the prevalence of both SUDs and mental health disorders but also by restricting the access of people in need of treatment to health services [4].

We are seeking papers that report new measures or that describe improvements to existing methodologies taken within health systems to improve care and care access of individuals with SUDs and associated comorbidities (e.g., psychiatric/psychological, cardiovascular, respiratory, hepatic, sexually-transmitted infections). Potential contributions may include, but are not limited to: the work of health professionals and health policies that contribute to reducing stigma and discrimination, as a way to ultimately improve the access to health care services or the outcomes of such services, as well as gender inequalities in the access to health services for SUDs; promising new treatment approaches (e.g., gender-responsive interventions, new medication-assisted therapies); evidence-based practices to improve the implementation, effectiveness, and evaluation of the health services’ response to unprecedented challenges (e.g., a new pandemic) or in humanitarian emergencies; the importance of addressing mental health as a way to prevent and treat SUDs.

We expect that the papers published in this Collection will lead to a better understanding of the challenges and opportunities in this field while contributing to reducing the burden of health systems.

## Data Availability

NA.
